# The impact of mitochondrial impairments on sperm function and male fertility: a systematic review

**DOI:** 10.1186/s12958-024-01252-4

**Published:** 2024-07-17

**Authors:** Minoo Vahedi Raad, Amir Masoud Firouzabadi, Maryam Tofighi Niaki, Ralf Henkel, Farzaneh Fesahat

**Affiliations:** 1 Department of Biology & Anatomical Sciences, Shiraz University of Medical Sciences, Shiraz, Iran; 2 Reproductive Immunology Research Center, Shahid Sadoughi University of Medical Sciences, Yazd, Iran; 3 Department of Physiology, School of Medical Sciences, Shahid Sadoughi University of Medical Sciences, Yazd, Iran; 4 Health Reproductive Research Center, Sari Branch, Islamic Azad University, Sari, Iran; 5 LogixX Pharma, Theale, Berkshire, UK; 6 Department of Metabolism, Digestion and Reproduction, Imperial College London, London, UK; 7 Department of Medical Bioscience, University of the Western Cape, Bellville, South Africa

**Keywords:** Assisted reproductive technology, In vitro fertilization, Male infertility, Mitochondria, Mitochondrial DNA, Mitochondrial membrane potential, Pregnancy outcomes

## Abstract

**Background:**

Besides adenine triphosphate (ATP) production for sustaining motility, the mitochondria of sperm also host other critical cellular functions during germ cell development and fertilization including calcium homeostasis, generation of reactive oxygen species (ROS), apoptosis, and in some cases steroid hormone biosynthesis. Normal mitochondrial membrane potential with optimal mitochondrial performance is essential for sperm motility, capacitation, acrosome reaction, and DNA integrity.

**Results:**

Defects in the sperm mitochondrial function can severely harm the fertility potential of males. The role of sperm mitochondria in fertilization and its final fate after fertilization is still controversial. Here, we review the current knowledge on human sperm mitochondria characteristics and their physiological and pathological conditions, paying special attention to improvements in assistant reproductive technology and available treatments to ameliorate male infertility.

**Conclusion:**

Although mitochondrial variants associated with male infertility have potential clinical use, research is limited. Further understanding is needed to determine how these characteristics lead to adverse pregnancy outcomes and affect male fertility potential.

## Introduction

### Importance of sperm mitochondrial function in male fertility

Globally, about 8–12% of couples suffer from infertility after a year of regular, unprotected intercourse, and 50% of these cases are caused by a male component. Since sperm motility is a crucial requirement for maintaining male fertility, current studies are focusing on the mitochondria as an indicator of normal sperm functionality and fertility [1]. One of the main factors contributing to many cases of “seemingly” idiopathic male infertility is sperm mitochondrial malfunction [2]. Mitochondria are responsible for generating reactive oxygen species (ROS), calcium homeostasis, cell death, and the generation of adenosine triphosphate (ATP) in the process of oxidative phosphorylation [3–5]. It has been suggested that mitochondrial physiology is one of the main factors to maintain chromatin integrity and execute acrosome reactions [6]. Mitochondria contain their own circular DNA (mtDNA) which, due to the absence of DNA-binding proteins such as histones, is about 100 times more susceptible to damage and mutations by high amounts of ROS and matrix-free radicals than nuclear DNA (nDNA) which is protected by histones or, in the case of sperm, by protamines [7]. Additionally, considering that mtDNA replicates quickly and does not have a reliable proofreading system like the nuclear genome [8], the repair processes for mtDNA are much less effective than those for nDNA. Hence, the mutation rates of mtDNA are reported to be 10 to 17 times higher than for nDNA [9].

Evidence suggests that about 80% of the chromosomal aberrations in humans are of paternal origin [10, 11]. It is widely accepted that in most animals, mtDNA is inherited solely from the mother, with the paternal mitochondria being essentially undetectable after the four-cell stage in a fertilized embryo [12]. This pattern of inheritance is called “maternal inheritance” [13]. However, recent findings cast doubt on this theory as there are indications that extra mtDNA is transferred from fathers to their children [14].

### Mitochondrial inheritance

According to a study by Luo and coworkers (2013), maternal factors do not destroy the mitochondria of spermatozoa during early embryonic development [15]. In this specialized polymerase chain reaction (PCR) based study, the authors demonstrated that most motile sperm cells lose their mtDNA during their movement toward the oocyte. However, if sperm with remaining mtDNA entered the oocyte and formed a zygote, this mtDNA was not eliminated but could still be passed on to the progeny and could be detected in the placenta and other extra-embryonic organs [15]. Yet, in order to shield the embryo from the consequences of damage by paternal mtDNA, sperm mitochondria and mtDNA should be diminished in the early stages of embryonic development [16]. Eker et al. reported that sperm mtDNA is occasionally maintained after whole sperm are injected into the cytoplasm of the oocyte during intra-cytoplasmic sperm injection (ICSI), suggesting that the offspring may actually have their father’s mitochondrial DNA [17]. As a result of intracellular ubiquitination, sperm mitochondria are selectively proteolyzed during early embryonic development [18, 19]. Thus, the proteolytic degradation of sperm mitochondria in the early embryo may be the reason why sperm mitochondria can affect fertilizing potential and early post-fertilization development more than later-stage development [3].

### Correlation of mitochondrial dysfunction and infertility

The most accurate measure of mitochondrial activity and the state of the body’s energy supply is the mitochondrial membrane potential (MMP or Δψ). Reduced sperm motility and decreased sperm fertilizing capacity are associated with impaired mitochondrial activity as shown by a decrease in sperm MMP [20]. This finding suggests that oxidative phosphorylation, the primary ATP production pathway within mitochondria, is essential for powering sperm motility. However, the study acknowledges the potential role of glycolysis as alternative pathway for energy production. While oxidative phosphorylation likely generates most of the ATP within the mitochondria, glycolysis might play a crucial role in transporting the generated ATP along the sperm’s tail to fuel motility [20]. In addition, some other studies demonstrated an association of sperm mitochondrial dysfunction with recurrent pregnancy loss and the failure of assisted reproductive technology (ART) procedures [21]. Although in vitro fertilization (IVF) is a commonly used method to treat couples with male infertility, its outcome remains unsatisfactory [22]. One of the most critical elements impacting therapeutic outcomes is sperm quality [23]. The latest WHO manual (WHO, 2021) recommends using strict criteria for identifying a normal spermatozoon and gives the following precise definition of a normal spermatozoon: *‘The head should be smooth, regularly contoured and generally oval in shape. acrosomal region comprising 40–70% of the head area without large vacuoles, and not more than two small vacuoles. the post-acrosomal region should not contain any vacuoles.the midpiece should be slender, regular and about the same length as the sperm head. Residual cytoplasm is considered an anomaly only when in excess, i.e. when it exceeds one-third of the sperm head size. principal piece with uniform caliber along its length, must be thinner than the midpiece, and approximately 45 lm long (about 10 times the head length).’*. The success of ICSI, as the most frequently applied method, has led to the use of immature as well as defective sperms regardless of subcellular sperm dysfunctions [24]. Failure of oocyte activation is thought to be the most common reason for ICSI failure [25]. Consequently, paternal parameters such as the lack of motile sperm, oocyte activating factors, globozoospermia, sperm DNA damage, and technical issues with sperm injection can all contribute to fertilization failure with ICSI [26]. Different procedures in ART labs have also been suggested to negatively affect sperm mitochondrial health in different ways [27]. Given the conflicting data regarding the impact of sperm mitochondrial dysfunction on pregnancy outcomes and ART success [28, 29], this systematic review aims to critically evaluate existing literature. Our focus is on how a deeper understanding of mitochondrial dysfunction can inform the development of novel ART strategies to improve male infertility treatment. This analysis will encompass a meticulous appraisal of the strengths and limitations within the included studies, facilitating a more nuanced interpretation of the current evidence and pinpointing areas for future research.

## Methods

In our literature search, we included the newest original and review scientific literature in the English language, published as full manuscripts (not abstracts) through PubMed, Scopus, and Google Scholar from January 2012 until August 2023. The reporting of this systematic review was guided by the standards of the Preferred Reporting Items for Systematic Review and Meta-Analysis Statement [30]. The following keywords and phrases were used: Male infertility, mitochondria, pregnancy outcomes, assisted reproductive technology, in vitro fertilization, intracytoplasmic sperm injection, sperm quality, mitochondrial DNA, and mitochondrial membrane potential [30].

**Fig. 1:**
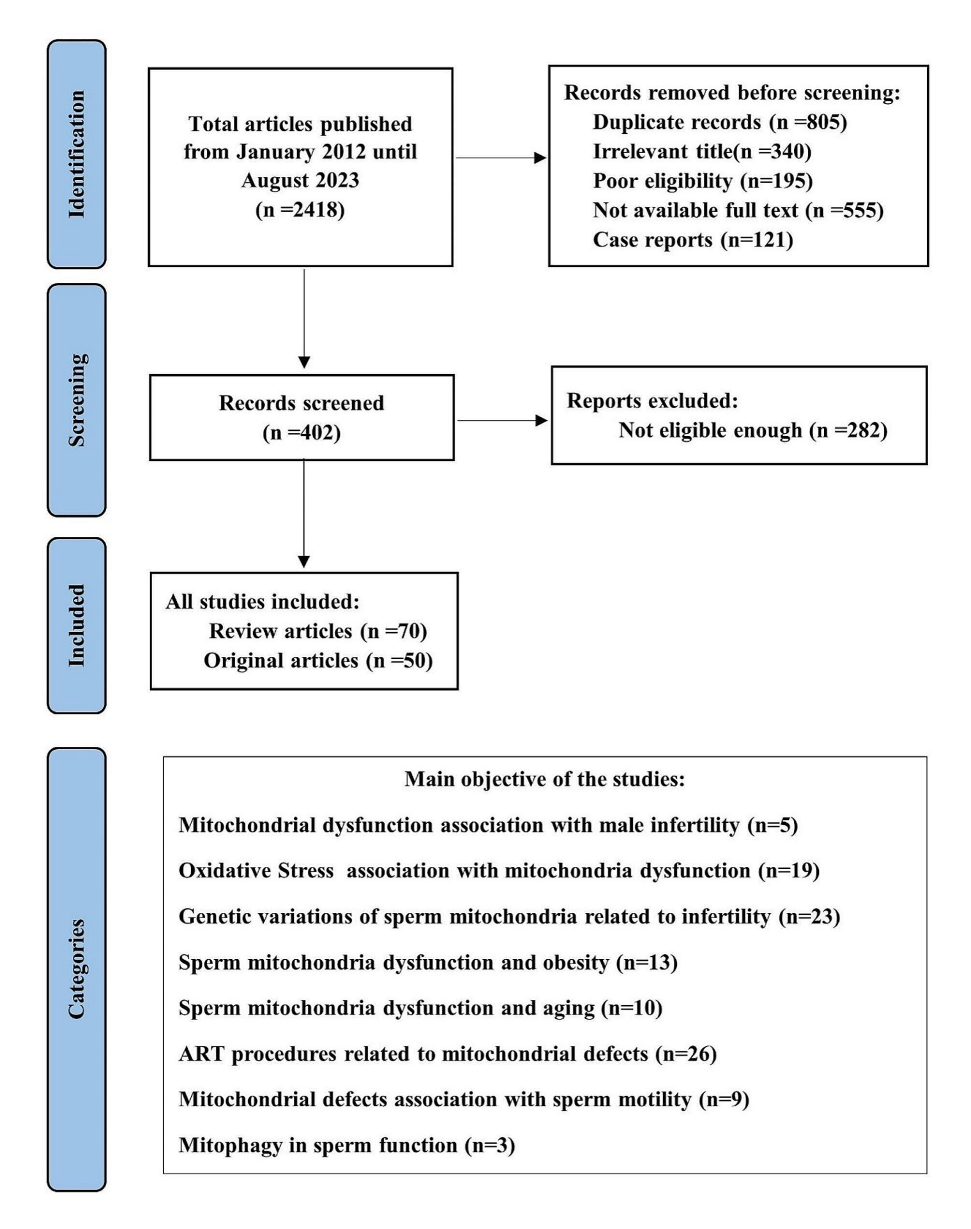
Flow chart of study selection according to PRISMA checklist

A total of 2418 articles were identified. After the first screening, 2016 articles were excluded as they were duplicated records or had poor eligibility for irrelevancy of the title or unavailability of the full text. Case reports were also excluded. A total of 402 articles (reviews and original research) were retrieved, of which 282 articles were excluded after full review (Fig. 1). The remaining 120 articles were selected as eligible for the present study: 70 of them were systematic and literature reviews, and 50 original articles.

## Results

Based on the studied articles, mitochondrial dysfunction and male infertility are strictly correlated. Mitochondrial impairment can be caused by factors such as oxidative stress, genetics, lifestyle, aging, and damaging ART techniques. Sperm mitochondrial dysfunction alters sperm function and can lead to poor success rates of ART procedures. Therefore, choosing the proper diagnosis tests is as important as selecting the right treatment option.

## Causes of sperm mitochondrial dysfunction

### Oxidative stress

Oxidative stress (OS) occurs as a result of an imbalance between elevated ROS levels and the antioxidant capacity in favor of the ROS. Elevated seminal ROS levels may be associated with idiopathic male infertility, thus being a potential screening tool in andrological diagnostics [31]. OS can be correlated with human semen quality, poor fertilization outcomes in ART procedures such as IVF and ICSI, impaired and slower embryo cleavage, poor blastocyst morphology, higher risk of embryonic death, and pre- and post-implantation losses [26, 32].

#### Sources of ROS in the male reproductive system

In the male reproductive organs, ROS are generated in several compartments such as the seminiferous tubules, epididymis, and in semen by leukocytes as well as to a lesser extent by sperm [33–35].

Any aerobic organism is producing its energy largely in the process of oxidative phosphorylation in the mitochondria. In this process, oxygen accepts electrons, thereby reduced to highly reactive intermediates, and water (H_2_O) as the final product is formed. However, during this physiological process, about 1–5% of the consumed oxygen is converted into ROS which are regarded as highly toxic byproducts and are linked to aging and many diseases including infertility [36–39].

Hydrogen peroxide (H_2_O_2_), superoxide ions (O_2_^•−^), and hydroxyl radicals (^•^OH) are the main ROS generated by cells with H_2_O_2_ mainly involved in redox sensing, signaling, and control [40]. In order to keep the levels of these highly reactive and therefore cytotoxic compounds in balance, antioxidants such as superoxide dismutase, glutathione peroxidase, catalase, glutathione reductase, ascorbate (vitamin C), and α-tocopherol (vitamin E), protect cells from these free radicals, so that the physiologic redox homeostasis is maintained [36]. Even during normal function, the mitochondrial electron transfer chain (ETC) loses electrons, reducing O_2_ to O_2_^•−^in a univalent manner. The mitochondrial superoxide dismutase converts 0.1–5% of the oxygen (O_2_) that is taken in by the cells into O_2_^•−^, which is subsequently quickly changed into H_2_O_2_ [41, 42]. Physiologically produced ROS at low levels play a crucial role in sperm motility, hyperactivation, transient sperm-oviduct adhesion, controlling sperm capacitation, and acrosome reaction [43, 44]. However, at higher levels ROS may have a negative impact on proteins, lipids, and the DNA, thus significantly harming cell functioning or triggering apoptosis [40, 45]. Hence, the physiological redox balance, homeostasis, must be maintained.

Other sources of ROS in semen include seminal leukocytes and immature spermatozoa. High quantities of immature spermatozoa exhibiting large amounts of cytoplasm, which is normally phagocytosed by Sertoli cells, result in increased ROS levels. A crucial defensive mechanism against pathogens, the myeloperoxidase system generates ROS from leukocytes in response to inflammation or infection in the male genital tract [46].

#### Effects of oxidative stress on sperm and mitochondrial function

ROS are produced from physiological metabolic reactions [47]. Even though several organelles including the plasma membrane, endoplasmic reticulum, and peroxisomes produce ROS in cells, mitochondria are the major source of ROS as they also regulate ROS production by non-mitochondrial sources [48–50] and are therefore the cell’s main endogenous source of ROS production [51]. When sperm ROS production surpasses the maximum amount of the natural antioxidative protective capacity, processes begin that can harm the sperm mitochondrial membrane and lead to mitochondrial dysfunction [52].

The mitochondrial electron transport chain (ETC) is composed of multiprotein transmembrane complexes (I–IV), generates an electrochemical gradient that enables complex V to synthesize ATP [53]. To function properly and produce ATP during oxidative phosphorylation (OXPHOS), these complexes must be assembled into a specifically configured super complex [41]. Endogenous mitochondrial ROS generation has been connected to the functioning of complexes I and III of the respiratory chain [54–56].

The presence of polyunsaturated fatty acids, which control the fluidity of the membrane and serve as favored targets for oxidative assaults by ROS, makes the sperm plasma membrane highly susceptible to OS-induced damage [57, 58], thus triggering lipid peroxidation and subsequently the formation of highly reactive lipid aldehydes, which create mitochondrial ROS and degrade sperm DNA integrity and competence by covalently attaching to ETC proteins [59]. Although mature sperm were previously considered as not being able to repair their DNA, recent research shows that the male germ cell has a truncated system for detecting and repairing DNA damage involving 8-oxoguanine DNA glycosylase (OGG1). This enzyme was shown to excise 8-hydroxy-2’deoxyguanosine (8OHDG) in the sperm nucleus and mitochondria. Yet, sperm do not possess the downstream enzymatic machinery to complete the repair making sperm more vulnerable to the accumulation of oxidative damage to mitochondrial and nDNA [59]. Additionally, sperm’s cytosolic area is constrained and contains few antioxidants after spermiogenesis [60].

### Genetic factors

#### Mitochondrial genome

Mitochondria have their own genome together with their transcriptional and protein-synthesizing machinery. The mitochondrial genome differs from the nuclear genome in several ways. Mitochondrial DNA is a circular, double-stranded genome that is 16.6 kb long. It contains the genetic material for 22 transfer RNAs, 2 ribosomal RNAs, and 13 polypeptide subunits necessary for the process of oxidative phosphorylation [61]. Within a single cell, there are hundreds to thousands of copies, and introns are not present. Few to no non-coding nucleotides connect its genes, and most of the time termination codons are absent. As highlighted before, due to the rapid replication of mtDNA and the absence of an effective modification mechanism, mtDNA modifications are predicted to be 10 to 17 times greater than that of nDNA (Table 1) [8]. On the other hand, they are produced post-transcriptionally by polyadenylation. Since there are multiple copies of mtDNA, the coexistence of mutant and wild-type molecules with heteroplasmy is possible [62].

**Table 1:** Human nuclear and mitochondrial genome differences

Feature	Nuclear DNA	Mitochondrial DNA	References
Location	Cell nucleus	Mitochondrial matrix	63–65
Size (in bp)	~ 3 × 10^9^	16,569	
Inheritance	Mendelian inheritance for autosomes and X chromosome; Paternal inheritance for Y chromosome	Maternal	
Structure	Double helix, linear, packaged in chromosomes	Double-strand, circular	
Cell division	Cell cycle regulated	Fusion/fission (continuous)	
DNA copies per cell	2 (1 allele from each parent)	~ 10–50,000	
Total DNA	99.75%	0.25%	
Number of genes encoded	~ 20,000–30,000 protein coding	13 protein-coding + 24 non-protein coding	
Replication pair	Yes	No	
DNA repair enzymes	Multiple	Polymerase gamma	
Gene density	~ 1 in 40,000 bp	1 in 450 bp	
Introns	Found in almost every gene	Absent	
Histones	Associated with the nuclear DNA	Not associated with the mitochondrial DNA	

#### The effects of mitochondrial DNA copy number on male fertility

The number of mitochondria differs in somatic cells [66]. Although the number of which is highly dynamic and regulated in a cell-specific manner by mechanisms that are not completely understood, each mitochondrion may contain 1 to 10 copies of the mitochondrial genome [67]. The mtDNA copy number is defined as the proportion of the mitochondrial genome copy numbers to nDNA copies [68]. The number of mtDNA copies in spermatozoa is only about 100. In contrast, oocytes have about 150,000 copies. For healthy sperm function throughout sperm growth and maturation, a reduction in mtDNA copy number is crucial [69].

Many studies have proven the association between increased mtDNA copy number and male infertility, whether it affects sperm parameters and lipid peroxidation in primary fertility or predicts the embryo development process [70–73]. All of these studies support the sensitivity of the mtDNA copy number to oxidative stress and its potential use as a biomarker indicating male fertility potential [70, 74]. It was also shown that infertile men with different sperm parameters have elevated mtDNA content [75]. According to the results of a study by Vahedi Raad et al. on the association between mtDNA and sperm parameters, which was done on two groups of infertile men with normal and abnormal sperm parameters and men who were fertile, this biomarker may be utilized to identify and perhaps cure general male infertility [71].

It is unclear how fewer copies of mtDNA would be beneficial for the development of sperms, but because oligozoospermic and asthenozoospermic males have very high levels of sperm mtDNA, scientists hypothesize that the likelihood of ROS-mediated damage is reduced by a decrease in mtDNA content in sperm [76, 77]. Nonetheless, there is no evidence to validate this hypothesis [73]. One hypothesis suggests that increases in the mtDNA copy number may be a healing mechanism by which oxidative damage or mtDNA deletions induce the reproduction of the mitochondrial genome [78]. The mitochondrial-nuclear crosstalk in the male germ cell, which must be kept in balance to drive vital sperm processes including sperm capacitation and sperm-oocyte fusion, becomes dysfunctional when there is excessive ROS production which can then lead to mitochondrial stress because the antioxidant enzyme systems may not be able to regulate the amount of ROS. In turn, this can modify the copy number of mtDNA resulting in mitochondrial DNA mutations, and disrupt the electron transport chain [79].

Atypical gene expression of mtDNA regulators like mitochondria-related mitochondrial transcription factor A (TFAM) or DNA polymerase γ (POLG) may be the cause of the increased mtDNA copy number in defective spermatozoa [80]. Since TFAM expression is normally down-regulated during spermatogenesis, greater levels of mtDNA copy number in aberrant spermatozoa may be related to a physiological aberration in this process [81]. Since the sperm mtDNA copy number was negatively correlated with urine polycyclic aromatic hydrocarbons (PAH) metabolite concentrations in young males, it is suggested that the sperm mtDNA copy number may be responsive to environmental exposures similar to how the leukocyte mtDNA copy number has been found to be affected by air pollution [70, 82–84]. Therefore, the evaluation of the sperm mtDNA copy number can be used as complementary data for male fertility potential assessment and may help to find the causes of idiopathic male infertility.

#### Mitochondrial gene variations related to male infertility

There are connections between mitochondrial abnormalities, sperm motility, fertilization rate, and full-term pregnancies because removing mitochondria from sperm and incubating them under oxygen in vitro reduced motility and viability. A significant accumulation of pathogenic mutant mtDNA in the testis results in a reduction in energy generation, ultimately causing meiotic arrest and changes in sperm morphology, thus emphasizing the significance of mitochondrial function in spermatogenesis [85].

The genes for *NADH dehydrogenase 1* (*ND1)* and *NADH dehydrogenase 5 (ND5)* are essential components of complex I [86]. Pathogenic variations in the NADH dehydrogenase (ND) genes are anticipated to impact complex I activity, resulting in a shortage in energy generation, which adversely influences sperm motility because complex I is essential for oxidative phosphorylation [87]. The mitochondrial genes ATPase6 and NADH dehydrogenase 4 (ND4) each have two single nucleotide polymorphisms (SNPs) that are related to asthenozoospermia at loci 9055 and 11,719, respectively [88]. Accordingly, the MT-ND4 gene missense mutation (11696G > A) causes the valine residue at position 313 to be replaced with isoleucine, thus changing the protein’s secondary structure. This variant has been attributed to decreased sperm motility [89].

An associated protein with cilia and flagella is encoded by the gene Cilia and Flagella Associated Protein 58 (CFAP58), which is primarily expressed in the testis [90]. CFAP58 is a protein that is found throughout the sperm flagella, but it is focused mainly on the middle section, which contains mitochondria. He et al. discovered an association between bi-allelic loss-of-function mutations in CFAP58 and common morphological abnormalities of the sperm flagella. Striking flagellar defects with malformed axonemal and mitochondrial sheaths were seen using transmission electron microscopy. Heat shock protein 60 (HSP60), a protein that forms the mitochondrial sheath, was significantly less abundant in the spermatozoa of males who carried bi-allelic CFAP58 variants, according to immunoblotting and immunofluorescence assays. Thus, CFAP58 deficiency can lead to axoneme and pre-axoneme abnormalities resulting in asthenoteratozoospermia [90].

Mutations and deletions of mtDNA are associated with male infertility [69, 91, 92]. Since mtDNA is present in hundreds to thousands of copies per cell, detecting mutant mtDNA and deletions does not indicate dysfunction as it is generally believed that the mutational load must surpass a threshold value (perhaps exceeding 60% of all mitochondria within a given tissue) for a notable phenotype [93]. The mtDNA 4,977 *bp* or common deletion, which appears in human mtDNA between nucleotides 8,470 and 13,447, is one of the most well-known mtDNA mutations [94]. This mutation eliminates all or a portion of the genes encoding the five tRNA genes, one complex IV gene, one complex V gene, two complex IV genes, and four complex I genes, all of which are necessary for maintaining normal mitochondrial function [95]. During aging, this deletion accumulates in various tissues and is linked to several illnesses. This has led to its adoption as a marker for mtDNA oxidative damage [96, 97]. The 4,977 *bp* deletion can be found in 40% of infertile men with mitochondrial dysfunction [98]. While several studies suggested an association between this genetic deletion and sperm parameters [99–101], some other studies could not find this association [72]. Since the frequent deletion of 4,977 *bp* is connected to aging and is only occasionally present in older males, contradictory additional studies are required for confirmation.

In addition to the typical 4,977 *bp* deleted mtDNA seen in infertile males, several earlier investigations also found the presence of deletions of 4,866, 4,856, 7,436, and 7,599 *bp* [92, 102, 103]. Ambulkar and colleagues suggested that the 7,436 *bp* deletions of mtDNA in spermatozoa may be one of the main causes of dysfunction and immotility of sperm [104]. The origin of these deletions remains debated [105].

### Lifestyle factors

#### Obesity

The prevalence of individuals with obesity is increasing in almost all countries [106]. Obesity and semen quality have both been examined in many studies [107–110]. Researchers found a connection between obesity and reduced mitochondrial activity, sperm progressive motility, and increased DNA fragmentation [111, 112]. Obesity is causing a systemic inflammatory response and is therefore associated with oxidative stress and inflammation [113–116] with elevated seminal ROS levels. The body mass index (BMI) is positively associated with seminal ROS levels [117].

According to an investigation by Binder et al., paternal obesity causes a delay in preimplantation embryo development starting at the second cleavage stage. These authors suggested that this delay is associated with embryonic genome activation. Accordingly, paternal obesity would damage the sperm, possibly at genetic or epigenetic levels [118, 119]. The authors also showed that the MMP in embryos derived from obese males’ sperm was significantly decreased as compared to normal sperm [118]. This delay could be due to the sperm from obese men being exposed to high oxidative stress levels as shown by Henkel et al. (not published). Although mitochondrial biogenesis does not occur during the preimplantation phase, mitochondrial DNA is actively transcribed starting at the 2-cell stage to produce respiratory chain components, which would contain proteins encoded by the father’s nDNA [120]. According to one study including 344 infertile couples, males with a BMI over 25 kg/m^2^ have a considerably reduced likelihood of clinical pregnancy success than men who are normal weight, however, day 3 embryo quality was unaffected [121]. Another study including 306 couples and 345 normal and overweight males revealed that the normal BMI group had significantly higher sperm activity, semen volume, and total sperm count than the overweight BMI group did. The relationship between obesity and increased oxidative stress, which may result in mitochondrial malfunction, is thought to be the cause of these findings [122]. Therefore, based on evidence from both animal and human models Campbell and co-workers suggested that improving the metabolic health of obese males can improve mitochondrial health and consequently male fertility [123].

#### Aging

Since it is evident that in recent decades couples tend to have children at older ages [124], it is crucial to understand how aging affects the fertility potential. The importance of maternal age for female fertility potential and reproductive outcome has been proven a long time ago [125]. However, later studies suggest that advanced paternal age is also associated with declined natural fertility, increased risk of spontaneous pregnancy loss, as well as the health of the offspring [126–131].

Mitochondria are regarded to be crucial to cellular activities that occur throughout the aging process. It is known that mtDNA mutations and mitochondrial dysfunctions increase with age in both animal models and in humans [132]. Although mitochondrial mutation levels increase with age, it is unclear if this increase plays a significant role in the process of aging [132].

The main reason behind mitochondria attribution in the aging process is ROS production [133, 134]. A study conducted by Kang and colleagues indicated that decreased citrate synthase function with age leads to male fertility decline. Citrate synthase is a vital enzyme of the mitochondrial tricarboxylic acid cycle, which directly regulates cellular function [135]. Age-related alterations in fatty acid content and decreased mitochondrial function have been identified in testicular mitochondria, may have an effect on the fluidity and activity of the mitochondrial complex, and are associated with a rise in the formation of superoxide anion, lipid peroxidation, and a decline in the activity of antioxidant enzymes [136–138]. Moreover, the number of Leydig cell mitochondria decreases in aging males [139], thus reducing the ability of Leydig cells to produce testosterone and steroid biosynthesis [140].

Usually, the female reproductive system eliminates defective sperm by natural selection and does not allow their fertilization with eggs. By using intracytoplasmic sperm injection (ICSI), all natural sperm selection barriers are bypassed, thus resulting in an increased risk of passing genetic defects to future generations [141], thereby older men may be more susceptible to this risk, as their ejaculates may contain more damaged sperm. Hence, better strategies should be developed to increase the number of good quality sperm after sperm selection for use in any ART process. Furthermore, in order to fully understand the real extent of the risks of fatherhood at later ages, it is also required to investigate the likelihood of genetic and epigenetic abnormalities in sperm from older men [139].

#### Lifestyle changes

Mitochondrial function can be negatively or positively affected by many natural dietary polyphenols, depending on the concentration. In addition, Astaxanthin present in seafood can increase MMP and improve sperm mitochondrial function [142]. Carbohydrates and proteins are also nutritional regulators of OS and testosterone levels and are firmly associated with sperm mitochondrial function [143]. Some other studies also suggested the cessation of smoking, especially hookah, reduces oxidative stress and decreases mitochondrial damage [144–146].

### Sperm preparation and cryopreservation techniques

In the andrology and embryology laboratory, oxidative stress levels may increase as the procedures such as centrifugation, manipulation of gametes under atmospheric oxygen tension, exposure to light, incubation time, and the type of sperm preparation technique, and/or the type of handling and culture medium used, as well as volatile organic compounds, and culture media additives have been shown to cause additional ROS production [147–153]. The two most frequently employed methods for sperm separation are swim-up and density gradient centrifugation. While the swim-up technique relies on the ability of motile sperm to swim out of the seminal fluid or a pellet of sperm into the supernatant culture medium, density gradient centrifugation selects sperm with normal morphology depending on their specific density [154].

The primary objective of sperm preparation techniques is to separate motile sperm from seminal plasma that contains non-motile, apoptotic, morphologically defective, and dead sperm [155]. When compared with the raw semen, ROS levels may increase throughout the centrifugation stages of certain sperm preparation procedures. In comparison with the swim-up, the chance of picking a spermatozoon with numerous dysfunctions is greater in the post-density gradient [156]. In order to reduce the oxidative stress levels even further, recent sperm selection techniques attempt to mimic the physiological processes in the female reproductive tract by employing microfluidics to reduce sperm oxidative stress and improve DNA fragmentation and the number of top-quality blastocysts [157–160].

The goal of sperm cryopreservation is to preserve spermatozoa of men who need to do so before receiving treatments that affect their ability to conceive such as chemotherapy, radiation, orchiectomy, or vasectomy for future use in ART, and donor insemination programs [161]. Numerous studies report that cryopreservation causes mitochondrial injuries, leading to a decrease in sperm MMP and ATP production [162]. Since mitochondria are the most sensitive sperm organelle to cryopreservation [163], it has been suggested that changes in mitochondrial membrane fluidity caused by cryopreservation increase MMP and induce the release of ROS, which in turn damages the sperm DNA by causing single-or double-strand DNA breaks [164]. The major event during cryopreservation is the disruption of energy supply due to the formation and prolonged opening of mitochondrial permeability transition pores, leading to a sudden collapse of mitochondrial function and reducing sperm motility and the ability to fertilize oocytes [165]. According to recent research on cryopreserved bull sperm, the opening of the mitochondrial permeability transition pore brought about by intracellular Ca^2+^ increases cause anomalies in mitochondria. This process is also linked to changes in MMP, ATP content, ROS levels, and plasma membrane integrity [166].

During the normal cryopreservation process, ice crystals are formed inside the sperm causing approximately half of the sperm being lost through cell death and lysis. Thus, the quality of the sample after thawing is adversely affected [167]. Techniques to reduce this cellular damage are vitrification and ultra-rapid freezing of the sperm and are therefore widely employed in advanced reproductive medicine [168]. In comparison with conventional cryopreservation protocols, vitrification has been shown to significantly improve sperm motility, recovery of motile sperm, DNA damage, and mitochondrial activity [169].

## Effect of sperm mitochondrial dysfunction on fertilization

### The effect of mitochondrial defects on sperm motility

Sperm motility is essential for male fertility. In choosing the best course of therapy for an infertile couple, motility is a crucial consideration. A minimum of 5 × 10^6^ sperm must be collected from a semen specimen to recommend intrauterine insemination (IUI); when 2 to 5 × 10^6^ motile sperm can be recovered, IVF is indicated as a therapy; and when samples only yield up to 2 × 10^6^ motile spermatozoa, ICSI is advised [170]. It has been demonstrated that reduced sperm motility can decrease fertilization success and impact the development of early embryos, even if some research suggests that pregnancy or the euploidy rate of the acquired blastocysts in ICSI is not affected. Also, it might be the result of a decreased blastocyst genesis rate or a developmental halt at the cleavage stage (day 3 embryos) [171]. Choosing the most motile sperm is beneficial as sperm motility is positively associated with good sperm DNA integrity [172].

As sperm approach the oocyte, progressive motility, generated by transverse waves moving from proximal to distal along the sperm flagellum, is important. By this mechanism, the sperm is propelled to the oocyte by a hydrodynamic impulse created as a result. Closer to the oocyte, and especially important for the penetration of the oocyte, sperm capacitate and develop hyper-activated motility characterized by an increase in high amplitude, asymmetrical flagellar beat, and increased ATP production [173]. This flagellar movement is driven by ATP, which acts as a molecular motor [174]. The energy is aerobically generated and depends on the oxidative activity of the mitochondria. In light of this, understanding mitochondrial activity may reveal important information about sperm motility [175].

Unfortunately, despite the clear connection between mitochondria and sperm motility, the knowledge has not yet been efficiently applied in clinical practice. Mutations and deletions in the mitochondrial genome impair sperm motility, resulting in infertility [176] and numerous mitochondrial genome alterations have drawn attention to the significance of mitochondria in male fertility [73]. Kinesin light chain 3 (KLC3), for instance, connects mitochondria, and its failure to bind outer dense fibers results in abnormalities in the mitochondrial sheath, changing progressive motility and causing infertility [177]. Additionally, it has been proposed that KLC3, selenoprotein phospholipid glutathione peroxidase 4 (GPX4), and Golgi-associated PDZ- and coiled-coil motif-containing protein (GOPC) are required to preserve the link between outer dense fibers and mitochondria [178].

The bulk of the defective proteins found in poorly motile sperm were found to be of mitochondrial origin [179]. Some other studies showed that mitochondrial dysfunction is correlated with mitochondrial enzyme activities, oxidative imbalance, and abnormal MMP [175, 180–182]. Furthermore, it was discovered that all of the assessed mitochondrial respiratory complex activities directly and favorably correlate with spermatozoa motility [183], implying that the condition of asthenozoospermia could be caused by changes in how mitochondria operate [20].

### The effect of mitophagy impairment on sperm function

To preserve cellular homeostasis under external stress, autophagy has been preserved through the evolution of eukaryotes. Autophagy is classified into three types: macro-, micro-, and chaperone-mediated autophagy [184]. When organelles and proteins are degraded for the production of new macromolecules or as a source of energy, macroautophagy is initiated by a stimulus such as famine and hypoxia [185]. Microautophagy can occur when cytoplasmic material invades the lysosome directly [186]. When heat shock protein 70 (Hsp70) chaperone connects with the lysosome-associated membrane protein type 2a (LAMP-2 A) during chaperone-mediated autophagy, it recognizes proteins with the KFERQ motif causing the attached protein to be translocated into the lysosome and atrophy [187].

Embryogenesis, implantation, and pregnancy maintenance are all significantly influenced by autophagy. Scientists suggest that mitophagy (autophagy of mitochondria) is essential for the differentiation of sperm before fertilization and the removal of paternal mitochondria after fertilization. According to some theories, autophagy controls cell homeostasis during spermatogenesis, changing the proportion of germ cells to Sertoli cells in the testis [188]. For the mitochondria to reorganize properly, extra mitochondria in the residual bodies must be removed by mitophagy during spermatogenesis [189]. In the absence of mitophagy, damaged mitochondria accumulate and cause oxidative stress [190].

When excessive ROS are produced in damaged mitochondria and these mitochondria are not removed by mitophagy, the loss of mitochondrial membrane potential and the release of cytochrome c can trigger apoptosis [191, 192]. The typical indications of cell death, such as cell shrinkage, chromatin condensation and fragmentation, cell vacuolization, and the formation of apoptotic bodies, are not present in spermatozoa. Therefore, the beginning of autophagy in sperm can only be detected with apoptosis cell markers. The function of mitochondria could be negatively impacted by the presence of apoptosis markers in the mid-piece area. As a result, the ability of the male gamete to penetrate the oolemma weakens, which lowers the potency of sperm fertilization [193, 194].

**Fig. 2:**
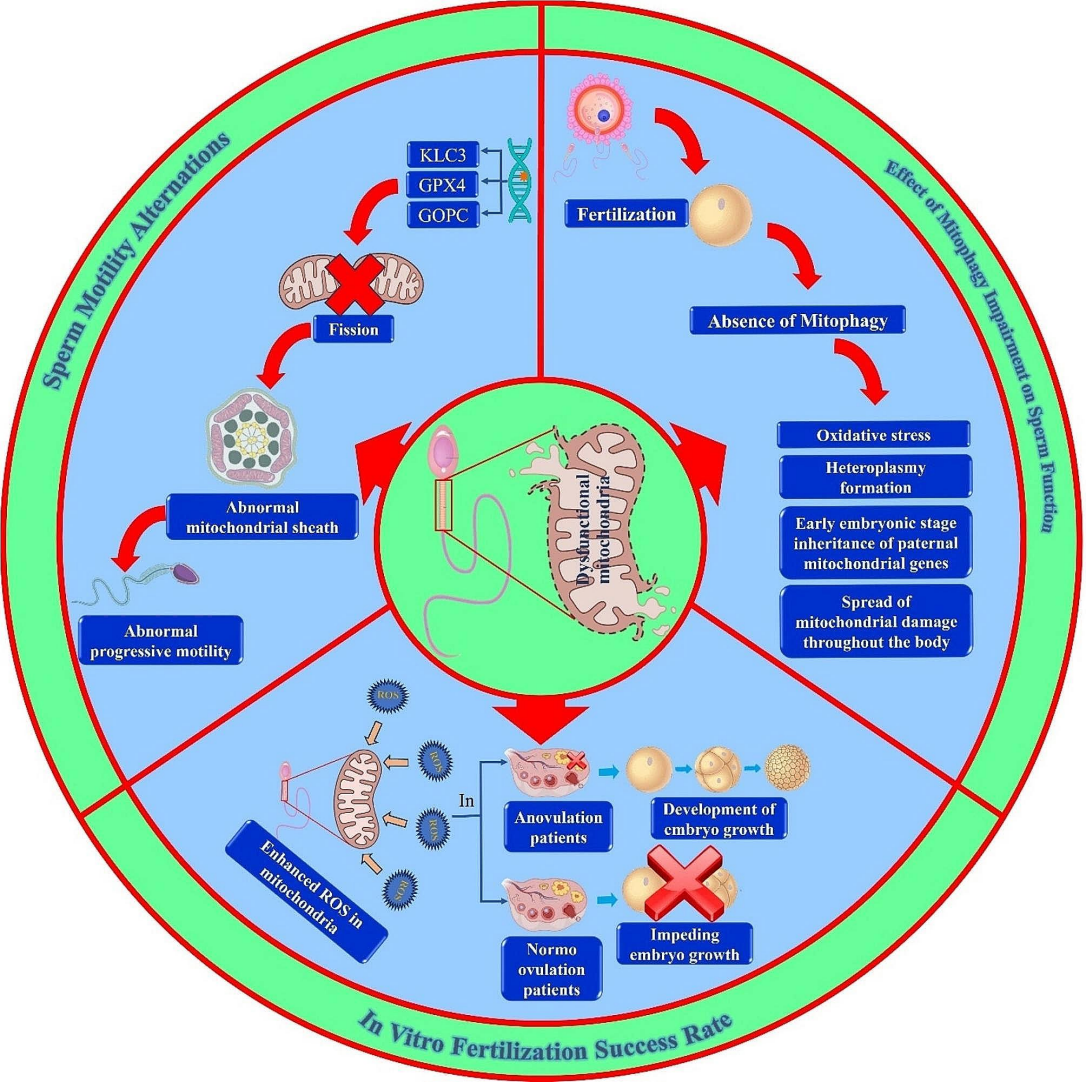
Some effects of sperm mitochondrial dysfunction on Fertilization GPX4: Selenoprotein phospholipid glutathione peroxidase 4, GOPC: Golgi-associated PDZ- and coiled-coil motif-containing protein, KLC: Kinesin light chain 3, ROS: Reactive oxygen species

Existing data is consistent with earlier predictions that sperm mitochondria penetrate the mammalian oocyte during fertilization and undergo active degradation inside the oocyte cytoplasm after fertilization [195]. In the majority of eukaryotic species, paternal mitochondria are destroyed by mitophagy through the autophagy receptors p62 and γ-aminobutyric acid receptor-associated protein (GABARAP) after fertilization (130). When autophagy is inhibited, heteroplasmy formation and the early embryonic stage inheritance of paternal mitochondrial genes occur [196]. Furthermore, mitochondrial damage can spread throughout the body due to the persistence of paternal mtDNA (132). Hence, the association between male infertility and mitophagy has significant clinical implications for future diagnosis and therapy (Fig. 2).

### Success rate of assisted reproductive technologies

Due to the broad use of ART technologies, especially ICSI, which bypasses all natural selection restrictions, accurate investigation to evaluate the health of the male germ cells is crucial in order to prevent the acute financial burden of repeated ART failure and to reduce emotional and psychological stress [197].

#### In vitro fertilization

Zhang et al. showed that even in normally looking sperm with poor fertilization rates after IVF, mitochondria might be impaired and dysfunctional [198]. Accordingly, sperm mitochondrial modifications can affect ART outcomes because of excess ROS production, mtDNA variations, mutations, deletions, decreased MMP, etc. Wu et al. reported that the sperm mtDNA copy number and mtDNA deletions are related to reduced probabilities of fertilization, high-quality Day 3 embryos, and transfer-quality Day 5 embryos in an ART environment [71]. Conceivably, important characteristics including smoking status and procedure type (IVF vs. ICSI) did not affect the outcomes [71]. Another study conducted by Yang and colleagues showed that paternal obesity results in lower sperm mitochondrial activity and poor IVF success rates [122].

Liu and colleagues reported that sperm mitochondrial ROS in cleavage-stage embryos have conflicting impacts depending on whether the female patient had normal ovulation. A high percentage of sperm producing a sufficient amount of ROS resulted in good development rates of day-3 embryos in anovulation patients, even though enhanced sperm mitochondrial ROS hindered embryo development in patients with normal ovulation [29]. This could be due to the fact that a small, physiological amount of ROS is necessary to trigger sperm capacitation, and acrosome reaction which are essential for embryo development [199–201]. Yet, further studies to investigate these mechanisms are necessary.

#### Intracytoplasmic sperm injection

Eker et al. discovered that male offspring after ICSI had the same SNPs in mitochondrial genes such as *cytochrome c oxidase subunit I (COX1)*, *ND1*, *NADH dehydrogenase 4 (ND4)*, and *ND5* as their fathers. In some cases, the degree of similarity reached up to 99% of the paternal mtDNA [17]. Tiegs et al. concluded that the sperm mitochondrial DNA copy number is not associated with fertilization, blastulation, blastocyst euploidy, and live birth rates after IVF and ICSI procedures [202]. In contrast, Wu and colleagues demonstrated that higher sperm mtDNA copy number and mtDNA deletions showed significantly lower odds of fertilization in ICSI treatment [71]. In another study conducted by Sharbatoghli et al., no significant correlation was observed between DNA fragmentation, apoptosis, and dysfunction of sperm MMP and ICSI outcomes [203]. According to research by Ghaleno and colleagues, large concentrations of internal H_2_O_2_ and O_2_•^−^in ICSI patients impact negatively the potential of the mitochondrial membrane. Nevertheless, only H_2_O_2_ might prevent pronuclear formation [204]. Such contradictory results highlight the urgent need for more detailed studies in this field (Fig. 2).

A study by Torra-Massana and colleagues demonstrated that five mitochondrial proteins have a higher abundance in patients with ICSI failure (dihydrolipoyl transacetylase (DLAT), D subunit of mitochondrial ATP synthase (ATP5H), solute carrier family 25 member 3(SLC25A3), solute carrier family 25 member 6 (SLC25A6), and fumarate hydratase (FH)). All these proteins are directly linked to the oxidative phosphorylation network [205]. Abnormal mitochondrial function may be linked to reduced proteasome system activity and vice versa. The authors suggested that sperm proteasomal activity is a factor that affects sperm fertilization ability in ICSI.

## Diagnostic tests and treatment of sperm mitochondrial dysfunction

### Diagnostic tests

Genetic differences and abnormalities in the mitochondrial genome can be detected by a variety of PCR methods. Furthermore, determination of the sperm energy metabolism of ATP levels is necessary. Measurement of ATP and ADP levels can be achieved using either high-performance liquid chromatography or commercial kits. Mitochondrial calcium levels can also be measured because Ca^2+^ is considered to be the primary regulator of oxidative phosphorylation. The evaluation of sperm calcium can be performed by fluorescent probes such as Quin-2 AM, fluo-3/AM, and indo-1 AM [206]. Other diagnostic tests are described below.

#### Mitochondrial activity index test

Nitro-blue tetrazolium is a useful indicator for testing the mitochondrial redox enzyme apparatus. In this test, the water-soluble, yellowish nitro-blue tetrazolium is converted into an insoluble blue formazan pigment in and around the mid-piece. In this method, sperm with normal mitochondrial activity shows a stained mid-piece, whereas poor staining is a marker of non-motile or poor mitochondrial activity sperm [207]. Some other indirect semi-quantitative tests in which the quantity of color generated would be indirect evidence of mitochondrial activity were also developed. The test was found to have a significant correlation with the sperm motility parameters [208].

#### Assessment of the mitochondrial membrane potential

Mitochondrial energy storage involves the proton gradients created by the inner mitochondrial membrane and electric potential across this membrane is casing the electron transport across the membranes for ATP synthesis [209].

Studies have shown a strong positive correlation between MMP and ROS production indicating that mitochondria produce more ROS at a high mitochondrial membrane potential [210, 211]. However, lower sperm motility and reproductive capacity are associated with altered mitochondrial activity, which is indicated by a decreased MMP [20]. The MMP is the most precise measure of mitochondrial energy state and function and is correlated with motility as a precise measure of sperm viability and fertilization capacity [212–215]. Due to the lower MMP in poorly motile sperm, it can be used as a factor for IVF success prediction because spermatozoa with low MMP are less capable of experiencing the acrosome reaction and a high percentage of motile spermatozoa is necessary for successful IVF [182]. Besides motility, sperm with high MMP are morphologically normal and can undergo acrosomal reaction. A high mitochondrial membrane potential does not necessarily mean the success of IVF, but if the potential is low, the process will fail and in this case, ICSI would be a more appropriate course of action [215]. Therefore, the determination of sperm MMP is a better criterion than sperm motility and can be recommended as a routine examination to assess sperm quality during diagnostic work-up for assisted reproduction as a supplement to the conventional semen analysis. To evaluate the MMP and sperm quality, fluorescent dyes like JC-1, MitoSOX Red, MitoTracker Green FM (MT-G), and MitoTracker Red CMH2XROS (MT-R) are utilized.

#### Measurement of oxygen consumption

This test directly assesses the ability of sperm mitochondria to utilize oxygen for energy production. Techniques like respirometry can measure the amount of oxygen consumed by sperm, indicating mitochondrial function. Ruiz-Pesini et al. showed that the mitochondrial membrane potential and oxygen consumption rate are positively associated with the ATP content, the proportion of motile sperm, and sperm velocity [216]. For the measurement of the oxygen consumption by human spermatozoa, after incubating the sample at 37 °C for at least 30 min, the assay plates are treated with concanavalin A, polyD-lysine, and Cell-Tak adhesive as coating materials to immobilize sperm cells on the bottom well. Next, to optimize the composition of assay media suitable for measuring mitochondrial respiration in human sperm, the XF Cell Mito Stress Test is performed with sperm cells plated in modified Tyrode’s solution [217].

### Treatment options for mitochondrial dysfunction

#### Antioxidant therapy

For the treatment of male infertility due to oxidative stress, the use of antioxidants is widely used [218]. The most often used formulations contain zinc, folic acid, N-acetylcysteine, co-enzyme Q10, vitamins E and C, selenium, carnitines, and pentoxifylline, all of which come in a variety of dosages and different combinations [175]. The quality of sperm is improved by supplementing the diet with unsaturated fatty acids such as ω-3 fatty acids, increasing mitochondrial energetic metabolism and decreasing OS [219]. Research has shown that the Mediterranean diet, which contains a high amount of fruits, vegetables, vitamins, proteins, and unsaturated fats, strongly affects sperm health and performance [220].

Selenium is essential for reproductive functions, including testosterone metabolism. It is also a component of the sperm capsule selenoprotein. The supplementation of infertile men with selenium significantly improved sperm motility. Selenoproteins have also been reported to maintain the integrity of sperm morphology [221]. Sperm antioxidant defenses depend on glutathione, which has also been found to improve sperm motility [222]. Selenium and glutathione are vital for phospholipid–hyperoxide GSH-Px formation which is an enzyme present in sperm cells. This enzyme becomes a structural protein with over 50% of the mitochondrial capsule in the mid-piece of mature spermatozoa. If either substance is deficient, the mid-piece may become unstable, leading to reduced motility [223].

Vitamin E is a crucial antioxidizing substance that primarily protects cell membranes by scavenging ROS and thereby preventing lipid peroxidation. This vitamin is thought to increase the synthesis of antioxidative enzymes that fight free radicals [223].

Since the conventionally used antioxidants are limited in their applications, a new methodology to formulate antioxidant treatments specifically targeting mitochondria as the source of ROS production has been developed. The lipophilic cation, triphenylphosphonium (TPP), is designed to target mitochondria and if conjugated with commonly used antioxidants, TTP-based antioxidants can reach 1000 times higher mitochondrial antioxidant concentrations [224]. These TTP-based antioxidants largely accumulate in pathological mitochondria to reduce oxidative stress in an MMP-dependent way [225]. Mitoquinone (MitoQ) is one of the well-known mitochondrial-targeted antioxidants. It has been reported that adding MitoQ to the thawing extender can improve sperm quality by decreasing ROS levels and preventing MMP loss in frozen sperm [226].

#### Assisted reproductive techniques improvement

Melatonin is an antioxidant that is typically produced in mitochondria and accumulated actively through the mitochondrial melatonin transporter. Mitochondria are both the main synthesis site and principal target for melatonin. Melatonin conserves MMP and maintains mitochondrial activities through rummaging of both ROS and RNS, preventing mitochondrial permeability transition pore opening. Furthermore, it functions as a signaling molecule that boosts the expression of genes involved in stress response and antioxidant enzymes [227]. This has led to the supplementation of cryo media with melatonin to avoid cryoinjuries brought on by mitochondrial abnormalities and OS during cryopreservation of the sperm. Melatonin can reduce the harmful reduction in the mitochondrial membrane potential and prevent the inhibition of mitochondrial oxidative phosphorylation and oxidative stress [228–230].

Myoinositol is the most abundant stereoisomer of inositol, influencing the intracellular Ca^2+^ concentration. Recently, Carlomagno and colleagues demonstrated that incubating sperm of oligoasthenoteratozoospermic (OAT) samples with myoinositol greatly enhanced their motility [231]. The enhancement of the intracellular sperm mitochondrial Ca^2+^ concentration due to their incubation in myoinositol containing medium may result in higher inner MMP in sperm cells, which is regarded as an apoptotic marker [232]. In patients with OAT, Rubino and colleagues reported higher motility and fertilization rate in the group where sperm were treated with myoinositol. Thus, myoinositol incubation of sperm might be used to identify patients who benefit from the incubation with myoinositol to increase motility [3].

## Conclusion

Spermatozoa rely on healthy mitochondria for ATP production which is essential for motility and fertilization. Mitochondrial dysfunction can lead to infertility. mtDNA damage and mutations can impact sperm function. An increased mtDNA copy number, antioxidants-ROS imbalance, disrupted mitophagy, obesity, and aging harms mitochondrial function leading to poor sperm motility and DNA integrity. Lifestyle changes like dietary modifications and cessation of smoking may improve sperm health. In assisted reproduction, techniques like centrifugation and cryopreservation can damage sperm mitochondria. Newer methods like microfluidics aim to minimize this damage. Evaluating sperm mitochondrial function, particularly through MMP assessment, can improve ART success after IVF and ICSI. However, further research is needed to understand the link between sperm mitochondrial dysfunction and ART outcomes, develop better methods to evaluate sperm health before ART, and explore therapeutic strategies targeting sperm mitochondria to improve male fertility. By optimizing sperm mitochondrial health, male fertility and success rates in ART procedures can potentially be improved.

## Data Availability

No datasets were generated or analysed during the current study.
